# Risk Factors of Delivery by Caesarean Section in Cameroon (2003-2004): A Regional Hospital Report

**DOI:** 10.5402/2011/791319

**Published:** 2011-11-03

**Authors:** P. M. Tebeu, E. Mboudou, G. Halle, E. Kongnyuy, E. Nkwabong, J. N. Fomulu

**Affiliations:** ^1^Ligue d'Initiative et de Recherche Active pour la Santé et l'Education de la Femme (LIRASEF), Yaoundé, Cameroon; ^2^Department of Obstetrics and Gynecology, Maroua Regional Hospital, Maroua, Cameroon; ^3^Department of Obstetrics and Gynecology, University Hospitals, Yaoundé, Cameroon; ^4^Department of Obstetrics and Gynecology, University Centre Hospital, Yaoundé, Cameroon; ^5^Reproductive Health Solutions, Salisbury, UK

## Abstract

We conducted this retrospective case-control study to identify possible risk factors of delivery through caesarean section in the Far North Region of Cameroon. Data was collected retrospectively from delivery room registers at the Provincial Hospital, Maroua, Cameroon from 01/01/2003 to 31/12/2004. The overall 125 eligible caesarean deliveries were compared with 244 women who delivered vaginally during the study period. The odds ratio as well as the 95% confidence interval was used to measure the relationship between maternal characteristic and risk of delivery by caesarean section. We found that the marital status is similar in the two study populations. Risk factors associated with cesarean section were: maternal age less than 17 years (OR 3.55, 95%CI: 1.46–8.64), maternal age over 39 years (OR 3.55, 95% CI: 1.17–10.75), nulliparity (OR 2.72, 95% CI: 1.59–4.66), grand multiparty (OR 3.43, 95% CI: 1.79–6.57), and macrosomia (OR 4.82, 95% CI: 1.49–16.44). There was a weak association with absent or poor. Caesarean delivery is associated with extreme ages of reproductive life, macrosomia, nulliparous and grand multiparous status. We strongly recommend that these factors be taken into consideration to strengthen the mother and child health programs in Cameroon and countries with similar socioeconomic profiles.

## 1. Introduction

Caesarean section may sometimes be the only means to save the life of the mother and/or foetus [1]. Current estimates in Cameroon put the national caesarean section rate at about 2%, with the lowest rate of 0.4% being reported in the Far North Region. This is lower than the national rate of 5–15% of the estimated live births, currently recommended by the United Nations [2]. A stillbirth rate of 7% to 12% was reported at the University Hospital, Cameroon [3, 4].

A recent study reported poor foetal outcome of fœtus delivered through caesarean section in Far North Cameroon Region and revealed that one of three caesarean deliveries ended up in foetal death [5]. A study in Nigeria revealed a higher perinatal mortality of 34% in women who refused elective caesarean delivery compared to 5% for those who accepted the procedure [6]. Refusal of caesarean delivery might be due to the lack of detailed information about the procedure. In order to perform caesarean section at right time for safety of the mother and her child, counseling on cesarean delivery must be part of each woman's prenatal care. The ultimate decision is based on the woman's obstetric history and the anticipated mode of delivery. The aim of this study therefore was to identify the possible risk factors of caesarean delivery that can be used as content of prenatal counselling in Cameroon.

## 2. Objective

We conducted this retrospective case-control study to identify possible risk factors of delivery through caesarean section that can be used as content of prenatal counselling in Cameroon.

## 3. Population and Methods

### 3.1. Population and Study Design

We conducted a retrospective case-control study of caesarean delivery outcomes in the Provincial Hospital, Maroua, Cameroon. Data was collected retrospectively from delivery and operating room registers between 01/01/2003 and 31/12/2004 inclusive. There were 144 cases of caesarean deliveries during the study period. After excluding cases of multiple pregnancies (*n* = 10) and cases where complete information was lacking (*n* = 9), we were left with 125 cases for analysis. After identification of each case of CS, data from the next successive 2 vaginal deliveries that followed were collected. Since it was possible to have consecutive CS before two vaginal deliveries, we finally compared 125 cases of CS with 244 cases of vaginal deliveries.

### 3.2. Variables

The maternal variables studied included marital status, number of prenatal visits, route of delivery, and maternal death. The neonatal variable was birth weight. Exact gestational age was not studied because it was not recorded in such cases. In Maroua, women are admitted for delivery irrespective of whether they have had prior antenatal care.

### 3.3. Statistical Analyses

The statistical software EPI-Info 3.4 was used for data analysis. The chi-square test was used to compare the distribution of the various variables in the two study populations. The odds ratio and the corresponding 95% confidence intervals were used to measure the relationship between mothers' characteristic and risk of delivery by caesarean section.

## 4. Results

The sociodemographic characteristics of women delivered by CS (*n* = 125) and those delivered vaginally (*n* = 244) are presented in Table 1. The marital status is similar among the two study populations. Compared to women delivered vaginally, women delivered by CS were more likely to be adolescents less than 17 years (6% versus 12%) or to be 40 years old and above (3.7% versus 7.2%).

The obstetric characteristics of the two study populations are presented in Table 2. Women delivered by caesarean section are more likely to be nulliparous (26% versus 40%), to be grand multiparous (13% versus 25%), and to have less than 4 prenatal visits (23.2% versus 31.6%) or no prenatal visit at all (2% versus 8%). The foetal weight at index delivery is presented in Table 3. Women delivered by caesarean section are more likely to have a foetus of 4000 grams and above (2% versus 8.8%).

The association between caesarean delivery and potential risk factor is presented in Table 4. Risk factors associated with cesarean section were maternal age less than 17 years (OR: 3.55, 95%CI: 1.46–8.64), maternal age over 39 years (OR: 3.55, 95%CI: 1.17–10.75), nulliparity (OR: 2.72, 95%CI: 1.59–4.66), grand multiparty (OR: 3.43, 95%CI: 1.79–6.57), and macrosomia (OR: 4.82, 95%CI: 1.49–16.44). There was weak association with absent or poor antenatal care.

## 5. Discussion

This study shows that the marital status is similar in the two study populations. The marital status in this study is consistent with the previous findings that almost all women who deliver in Far North Cameroon are married [7]. 

This study shows that when the parturient's age is less than 17 years, the rate of delivery by caesarean section is doubled and the risk of caesarean delivery is tripled compared to women aged 20–29. Unfer et al. documented a higher rate of caesarean section in teenagers compared to women in their twenties [8]. Contrasting findings were reported by others [9, 10]. Lao and Ho found that incidence of caesarean section was lower in the teenage mothers [10]. They attributed the good results observed to the free and readily available prenatal care and the quality of support from the family or welfare agencies that were involved with the care of teenage mothers. However, these studies had some limitations; they included all teenagers until 19 years and are therefore a heterogeneous population [9, 10]. In the present study the proportion of teenagers aged 17–19 is similar in both study populations (19% versus 20%).

We found that women delivered by CS were more likely to be aged 40 and above (3.7% versus 7.2%) and the risk of caesarean delivery is more than threefold when compared to women aged 20–29 years. In a previous study at the University Centre Hospital, we found that the risk of delivery by caesarean section for women in their forties (16.1%) was significantly higher compared to women in their twenties (10.0% versus 16.1%) HR: 1.7; 95%CI: 1.1–2.8; *P* = 0.027) [11]. Other studies found an increased risk of caesarean delivery in nulliparous and multiparous women aged 40 years and above [12, 13]. the grand multiparity observed in caesarean delivery group of our study (13% versus 25%) could have contributed to the risk of caesarean delivery.

Women delivered by caesarean section are more likely to be nulliparous (26% versus 40%) and the risk of caesarean delivery is about 3 times for nulliparous women compared to those with parity of 1–5.

Grand multiparous women were mostly in the caesarean delivery group (13% versus 25%), with the caesarean delivery risk increased by 3 times among grand multiparous women. Nulliparous women in Maroua are more likely to be teenagers aged less than 17 years and multiparous women are more likely to be 40 and above. Both conditions are known risks of caesarean delivery [8, 11].

Women delivered by caesarean section were more likely to have had less than 4 prenatal visits (23.2% versus 31.6%) or no prenatal visit at all (2% versus 8%). However, only a weak association was found between the antenatal care and the risk of caesarean delivery. This observation underscores that the ANC is not effective as such in preventing cesarean delivery in the Far North Region of Cameroon. Our findings corroborate with results from the MOMA group who failed to identify a strong ANC parameter to predict the occurrence of dystocia in West Africa [14]. However, in spite of the weakness of the positive predictive value, there is a close relationship between severe maternal morbidity and many sociodemographic and obstetric characteristics of the patients [15]. In the present study the frequency of ANC care was about one visit per month. This could lead to overload of work which in turn limits the possibility of health workers to correctly identify potential risk factors and ensure the continuity of the care till the delivery. However, studies from West Africa revealed a high incidence rate of dystocia (18%) and severe maternal morbidity (6.7%) and underscore the need to be alert during ANC and delivery. Ten risk factors for severe maternal morbidity were identified, and health workers need to be aware of them during delivering ANC. These risk factors are hemorrhage during pregnancy, antecedent of CS, high blood pressure, antecedent of multiple pregnancy, height less than 150 cm, lack of fetal movements, history of stillborn, age over 35 years, nulliparity, and presence of disease during pregnancy. 

Pregnant women require knowledge on caesarean delivery. The antenatal visit is the right moment to provide the counselling related to this mode of delivery. The basic component of the new World Health Organization (WHO) antenatal care model prescribes reduced number of clinic visits at 4 and limited investigations for low-risk pregnant women [16, 17]. The inadequate antenatal care in our population could have influenced the caesarean delivery because the ANC is also the opportunity to anticipate macrosomia and induce labour before the expected delivery date and avoid excessive foetal growth.

The current study has some limitations. First, this is a retrospective study and is therefore subjected to some information bias. In addition, due to the limited number of cases in different categories, we did not perform adjusted analysis. However, this study provides baseline information on the topic and gives the opportunity for further prospective studies.

## 6. Conclusion

Caesarean delivery in Cameroon is associated with extreme ages of reproductive life, macrosomia, nulliparous and grand multiparous status. These findings suggest that some of the caesarean section cases could be avoided while others could be anticipated. The study provides relevant information for counselling of pregnant women on timely caesarean section when indicated. We strongly recommend that these issues be taken into consideration in order to strengthen the mother and child health programs in Cameroon.

## Figures and Tables

**Table 1:** Sociodemographic characteristics according to the mode of delivery.

Characteristics	Caesarean delivery			*P*
	Yes	No	Total	
	*N* = 125	*N* = 244	369	
	*N* (%)	*N* (%)	*N* (%)	
Age classes (years)				
20–29	33 (26.4)	117 (48.0)	150 (40.7)	0.000
17–19	24 (19.2)	49 (20.1)	73 (19.8)	
<17	15 (12.0)	15 (6.1)	30 (8.1)	
30–39	29 (23.2)	47 (19.3)	76 (20.6)	
≥40	9 (7.2)	9 (3.7)	18 (4.9)	
Not available	15 (12.0)	7 (2.9)	22 (6.0)	
Marital status				
Married	119 (95.2)	234 (95.9)	353 (95.7)	0.754
Single	6 (4.8)	10 (4.1)	16 (4.3)	
Year of delivery				
2003	44 (35.2)	92 (37.7)	136 (36.9)	0.637
2004	81 (64.8)	152 (62.3)	233 (63.1)	

% percentage; *P* significance.

**Table 2:** Obstetrical characteristics.

Characteristics	Caesarean delivery			*P*
	Yes	No	Total	
	*N* = 125	*N* = 244	369	
	*N* (%)	*N* (%)	*N* (%)	
Parity				
1–5	42 (33.6)	144 (59.0)	186 (50.4)	0.000
0	50 (40.0)	63 (25.8)	113 (30.6)	
≥6	31 (24.8)	31 (12.7)	62 (16.8)	
unkown	2 (1.6)	6 (2.5)	8 (2.2)	
Antenatal care attendance				
4 and above	29 (23.2)	77 (31.6)	106 (28.7)	0.043
1–3	43 (34.4)	99 (40.6)	142 (38.5)	
0	13 (10.4)	17 (7.0)	30 (8.1)	
Not available	40 (32.0)	51 (20.9)	91 (24.7)	

%: percentage; *P*: significance.

**Table 3:** Foetal weight at index delivery.

Characteristics	Caesarean delivery			*P*
	Yes	No	Total	
	*N* = 125	*N* = 244	369	
	*N* (%)	*N* (%)	*N* (%)	
*Weight of the neonate (grams)*				
2500–3999	94 (75.2)	206 (84.4)	300 (81.3)	0.009
2000–2499	12 (9.6)	21 (8.6)	33 (8.9)	
<2000	2 (1.6)	8 (3.3)	10 (2.7)	
≥4000	11 (8.8)	5 (2.0)	16 (4.3)	
Not available	6 (4.8)	4 (1.4)	10 (2.7)	

%: percentage; *P*: significance.

**Table 4:** Risk of caesarean section according to the characteristics of mother and foetus.

Potential risk factor for caesarean delivery	Caesarean delivery (*N*)	Vaginal delivery (*N*)	Total (*N*)	Crude odds ratio (95%CI)	*P* value
Age of the patient					
20–29	33	117	150	1 ^a^	
<17	15	15	30	3.55 * (1.46–8.64)	0.0015
≥40	9	9	18	3.55 * (1.17–10.75)	0.0095
Parity					
1–5	42	144	186	1 ^a^	
0	50	63	113	2.72 * (1.59–4.66)	0.0000
≥6	31	31	62	3.43 * (1.79–6.57)	0.0000
ANC					
≥4	29	77	106	1 ^a^	
0	13	17	30	2.03 (0.8–5.09)	0.094
1–3	43	99	142	1.15 (0.64–2.09)	0.615
Foetal weight (grams)					
2500–3999	94	206	300	1 ^a^	
≥4000	11	5	16	4.82 * (1.49–16.44)	0.0019

^a^Reference category; *Significant.
